# Metrological and Critical Characterization of the Intel D415 Stereo Depth Camera

**DOI:** 10.3390/s19030489

**Published:** 2019-01-25

**Authors:** Monica Carfagni, Rocco Furferi, Lapo Governi, Chiara Santarelli, Michaela Servi, Francesca Uccheddu, Yary Volpe

**Affiliations:** Department of Industrial Engineering, University of Florence, 50139 Firenze, Italy; monica.carfagni@unifi.it (M.C.); lapo.governi@unifi.it (L.G.); chiara.santarelli@unifi.it (C.S.); michaela.servi@unifi.it (M.S.); francesca.uccheddu@unifi.it (F.U.); yary.volpe@unifi.it (Y.V.)

**Keywords:** reverse engineering, RealSense D415, depth camera, device characterization, VDI/VDE standard, active stereo, performance comparison

## Abstract

Low-cost RGB-D cameras are increasingly being used in several research fields, including human–machine interaction, safety, robotics, biomedical engineering and even reverse engineering applications. Among the plethora of commercial devices, the Intel RealSense cameras have proven to be among the most suitable devices, providing a good compromise between cost, ease of use, compactness and precision. Released on the market in January 2018, the new Intel model RealSense D415 has a wide acquisition range (i.e., ~160–10,000 mm) and a narrow field of view to capture objects in rapid motion. Given the unexplored potential of this new device, especially when used as a 3D scanner, the present work aims to characterize and to provide metrological considerations for the RealSense D415. In particular, tests are carried out to assess the device performance in the near range (i.e., 100–1000 mm). Characterization is performed by integrating the guidelines of the existing standard (i.e., the German VDI/VDE 2634 Part 2) with a number of literature-based strategies. Performance analysis is finally compared against the latest close-range sensors, thus providing a useful guidance for researchers and practitioners aiming to use RGB-D cameras in reverse engineering applications.

## 1. Introduction and Background

Three-dimensional optical systems have found popularity in numerous fields of application spanning from robotics [1], automotive [2], industrial [3], mechanical engineering and cultural heritage [4,5], to the biomedical field [6,7,8]. Their success is mainly due to recent developments, which have allowed the creation of devices that are less expensive, yet accurate and compact.

Microsoft’s original Kinect hardware was powered in September 2009 by PrimeSense [9]. The Israeli company pioneered the technology of projecting an infrared (IR) dot pattern onto a scene and detecting the dots with an IR camera to assess depth information. The output of the Kinect in its first version was a 320 × 240 depth map with 2048 levels of depth values, based on the projected IR speckle pattern. Later, other companies released low-cost Kinect-like depth cameras (i.e., Asus Xtion [10], Astra Pro 3D [11], and Occipital Structure Sensor [12]). As solely human natural interface tools, such sensors were widely regarded as unsuited for gaming, nevertheless, the revolutionary depth-sensing technology provided a significant boost for robotics and machine vision [13].

In 2013, Apple bought PrimeSense and the depth camera technology continued to evolve. The Kinect v2 for the Xbox One replaced the PrimeSense technology with Microsoft’s own time-of-flight technology [14], resulting in a much higher accuracy and resolution. In 2016, Lenovo launched the Phab 2 Pro, the first smartphone to implement Google’s Tango technology for augmented reality and machine vision, and which was also based on infrared depth detection [15]. In late 2017, Apple released the iPhone X including a Kinect-like miniature depth sensor [16]. Unlike the original Kinect, which was built to track motion in a whole living room, the sensor is primarily designed for scanning faces and powers Apple’s Face ID feature [17].

Meanwhile, Intel also built its own depth sensor family, Intel RealSense [18], and in 2015, worked with Microsoft to power Windows Hello, a 3D face recognition means to sign in to Windows 10 devices [19].

Despite the fact that Intel RealSense devices have only appeared on the market in recent years, they have been adopted in several fields. Among the many fields and applications, we can find posture and gesture interaction systems and human interaction design [20,21,22,23,24], interactive artificial intelligence (AI) toys for children [25], security and robotics [26,27,28], and also medical and human care [29,30,31].

RealSense technology basically consists of vision processors, depth and tracking modules, and depth cameras, supported by an open source multi-platform software development kit (SDK) called *librealsense* [32] that simplifies camera support for software developers and third-party system integrators. To overcome previous camera releases and strengthen their leading position in the market, Intel launched two new depth cameras in January 2018: the D415 and the D435 models. Such devices differ from each other mainly in the field of view (FOV) angles and in the exposition time of the camera-integrated shutter. The larger FOV of the Intel RealSense D435 depth camera translates into a minimization of blind spots, thus making it better for use cases such as robotics; the global shutter provides better performance when capturing high-speed movements, avoiding depth image blurring, or shooting in low-light situations. Having a smaller FOV, the Intel RealSense D415 has a higher pixel density, thus resulting in a higher resolution. Thereby, when accuracy is paramount (e.g., for 3D scanning applications), the Intel RealSense D415 promises to provide better results, especially when used at a short range (i.e., <1 m).

Since the use of RGB-D cameras as a low-cost 3D scanner has spread across many applications, a comprehensive characterization of this new device is needed to identify the best device and its parameter settings for each scanning scenario. In a recent work, Giancola et al. [33] proposed characterizations for the Microsoft Kinect v2, the Orbbec Astra S and the Intel D400 series. For each of these devices, two types of experiments were performed, one for pixel-wise and one for sensor-wise characterization to evaluate, respectively, the accuracy of the cameras at different distances and the quality of the reconstruction of known geometries. To evaluate the quality of the reconstruction of known geometries (i.e., sensor-wise characterization), the distribution of the distance between the acquired points and the actual geometric models was measured; during these tests, known planes, cylinders and spheres were acquired. The pixel-wise characterization of both the Microsoft Kinect v2 and Orbbec Astra S cameras was done by placing each camera on a photographic tripod and aligning it with a white planar target mounted on an anthropomorphic robot with an arm reach of 1200 mm, and repeating the test four times to cover a distance from 800 mm to 4200 mm. As for the pixel-wise characterization of the Intel D400 cameras, the setup involved the use of two coordinated robots. The camera was fixed on one of the two robots and the target on the other, thus allowing the entire range to be covered in a single test (e.g., without moving the setup). The author of Reference [33] reported, as expected, that the best depth camera performance (in terms of pixel-wise accuracy and accuracy of the reconstruction of known geometries) is at the closest working range.

To pave the way towards a global performance measurement standardisation, thus providing users with a comprehensive analysis of camera limits and strengths in the best case of a close-range 3D scanner application scenario, additional tests are needed. Unfortunately, at the present time, the international community has not yet released a recognized standard for non-contact 3D imaging systems that includes depth camera systems.

The German standard VDI/VDE 2634 Part 2 (“Optical 3D measuring systems”) [34] is, in fact, the only recognised standard providing a number of guidelines for the acceptance and verification of optical 3D measuring systems. It consists of a set of acceptance tests mainly based on evaluating three quality parameters: probing error, sphere spacing error, and flatness. Measurements are carried out using prescribed artefacts including a sphere, ball bar, and flat object. The guidelines indicate the exact size of the known artefacts according to the acquisition volume; in particular, the artefact’s size is related to the length L_0_ of the diagonal of the 3D imager’s conic field of view (as explained in Section 3). For each of these objects, both an acceptance test (i.e., calibrated artefacts are measured) and a re-verification test (i.e., the test is repeated over time) are uniquely defined. The tests are accepted if the error lies inside the limits specified by the manufacturer. Three errors are introduced by the standard: (1) the probing error, P, which describes the characteristic error of the system within a small part of the measurement volume; (2) the sphere spacing error, SS, which demonstrates the ability of the system to measure lengths; and (3) the flatness measurement error, F, i.e., the range of the signed distances of the measured points from the best-fit plane. Based on such a standard, in a previous work [35], the authors proposed a metrological and critical characterization for the previous RealSense RGB-D camera, the Intel SR300. The proposed methodology for the sensor characterization was delivered by integrating VDI/VDE guidelines with the recent characterization strategies provided in the scientific literature [33,36,37,38].

Inspired by such a work, which allowed a full description of the metrological properties of the SR300, the main aim of this paper is to characterize and to provide metrological considerations for the Intel RealSense D415 depth sensor, taking into account both current professional standards and best practices derived from the literature.

Additionally, the performance of the D415 is compared against the RealSense SR300 and other recent short-range devices (i.e., the PrimeSense Carmine 1.09 and Kinect v2), thus feeding the current critical discussion on this category of devices. This will allow researchers and practitioners to choose the optimal device for their own reverse engineering applications.

This paper is organized as follows. Section 2 presents the Intel RealSense D415 depth camera specifications and working principles. The devised test set is presented in Section 3. Finally, experiments are discussed in Section 4 and the conclusions are presented.

## 2. Intel RealSense D415 Depth Camera

The Intel RealSense Depth Camera has been designed to equip devices with the ability to see, understand, interact with, and learn from their environment. The D415 features the Intel RealSense Vision D4 processor with a high-resolution depth (up to 1280 × 720 pixels at 30 frames per second), long-range capability (up to about 10 m), rolling shutter technology and, as noted, a narrow field of view ideal for precise measurements. The RealSense Vision D4 is a vision processor based on 28-nanometer (nm) process technology for real-time calculation of stereo depth data.

**Figure 1:**
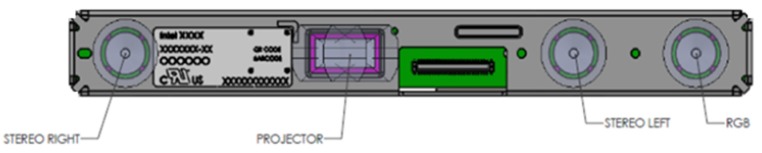
The component locations inside the RealSense D415 camera.

The device has a very compact depth camera (dimensions: 99 × 20 × 23 mm^3^, weight: 72 g) that can either be integrated into computers and mobile devices or used as a self-standing device. Moreover, it comes with a color camera and a depth camera system, comprising two IR cameras and an IR projector (Figure 1).

**Figure 2:**
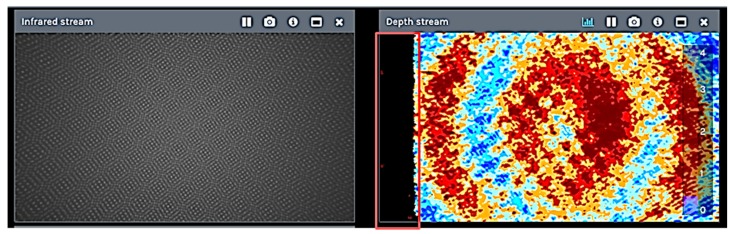
The static infrared dot pattern projected on a wall (**left**); highlighted in red is the non-overlapping region on the field of view of the left and right image (**right**).

The infrared projector improves the ability of the stereo camera system to determine depth by projecting a static infrared pattern (Figure 2) on the scene to increase the texture of low-texture scenes. The left and right imagers capture the scene and send raw image data to the vision processor, which calculates depth values for each pixel in the image by correlating points on the left to those on the right image. The depth pixel values are processed to generate a depth frame. This active stereo depth computation makes this depth camera suitable for acquisitions both indoors and outdoors under reasonable illumination. To this purpose, the IR projector intensity can be tuned manually according to the environmental lighting conditions. The depth data generated with stereo vision uses the left image as the reference for stereo matching resulting in a non-overlapping region on the field of view of the left and right image. Therefore, there is no depth data at the left edge of the frame (Figure 2 in red). Closer scenes result in a wider invalid depth band than scenes at further distances.

**Figure 3:**
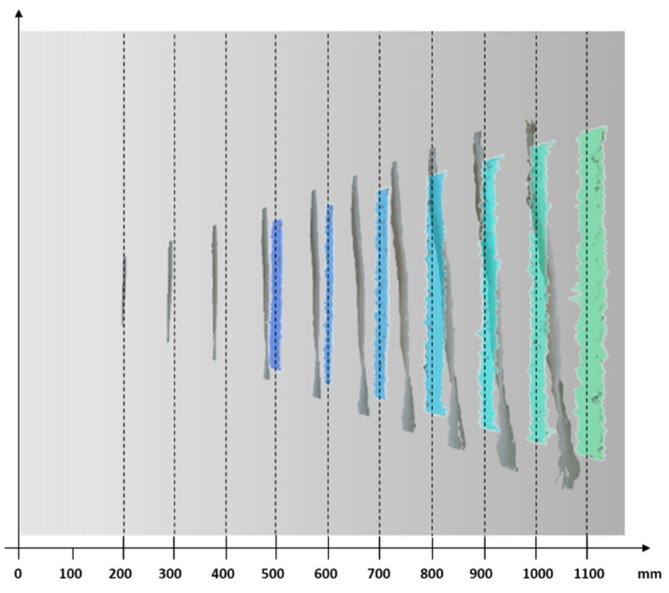
The qualitative evaluation of the error introduced in measuring a flat surface, and comparison between SR300 (grey point clouds) and D415 (colored point clouds) models.

The SR300 and the new D415 have different working principles for depth measurements (i.e., active stereo vs. structured light), which results in different performance as depicted in Figure 3. The figure shows a qualitative comparison of the two devices in representing a flat surface acquired at different camera distances. In grey are depicted the point clouds of a plane acquired at increasing distance using the SR300 model; the plane acquired with the D415 camera is instead described by the colored point clouds. It can be observed that the new device has a considerably lower error both in terms of planarity and of distance accuracy.

The D415 has a narrow field of view solution using rolling shutter sensors; this means that very fast-moving objects or the fast movement of the device (such as quick horizontal panning) can result in slightly distorted images. The Intel RealSense D400 series is supported by the cross-platform and open source Intel RealSense SDK 2.0 [32], a library that allows the configuration of the camera with several internal settings (both for acquisition and post-processing). Furthermore, a set of visual pre-sets to improve performance is available. Such versatility allows users to arrange the best possible setup for the task under investigation.

The D415 is equipped with a color camera with a resolution up to 1920 × 1080 pixels, and provides texture information to be superposed on the depth data. The spatial resolution of the depth map of the Intel RealSense D415 is up to HD (1280 × 720 pixels), in a working depth range declared by the manufacturer equal to ~160–10,000 mm. The camera works at different pixel resolutions corresponding to different minimum depth values, point densities and framed areas. The specifications reported in Table 1 refer to the HD format. The declared horizontal field-of-view (FoV) (both for the depth and the RGB cameras) is approximately 69° and the vertical FoV is approximately 42°. The baseline between the two IR cameras is 55 mm. As can be seen from Table 1, which summarizes the technical characteristics of the device, horizontal and vertical FOVs are subject to an error of ±3 degrees. For this reason, the point density and framed area variations, evaluated by increasing the distance between the camera and the acquired scene are reported in Table 2, Table 3 and Table 4. Point density variation is evaluated, respectively, for the FOV as indicated in the Intel specifications (69.4 × 42.5°, Table 2), for the two FOV extrema (i.e., obtained by removing and adding 3°, Table 3), and the actual FOV of the particular camera used to carry out the performance analysis (67.24 × 41.01°, Table 4). The FOV of the experiment camera was evaluated on a depth frame with the *get_stream_intrinsics* function implemented in the librealsense SDK.

## 3. Materials and Methods

As mentioned above, the device under examination can work in different configurations. The camera software interface supports several predefined depth presets that can be selected according to the user’s usage. Among the available presets, the *Default* configuration provides the best camera parameters to obtain the best visual appeal, clean edges, and to reduce point cloud spraying. With the aim of providing a comprehensive characterization of the camera and to enable a comparison with the other similar devices, while keeping the analysis as general as possible, in this work, the *Default* configuration was considered as the starting point from which to vary only a subset of critical parameters.

The characterization of the device was carried out by considering the maximum IR camera resolution (i.e., 1920 × 1080 pixels) and in a working range lower than 1 m (short range). Furthermore, tests were performed entirely by setting the depth unit (i.e., the depth step size) to its maximum value, equal to 100 µm, to obtain the best possible depth quantization. This limited the maximum range of the camera to circa 6.5 m. Lower depth unit values were not adopted to avoid quantization effects.

The critical parameters which were varied within the experiments are the disparity shift and the laser power, the former influencing the possible working distance and the latter influencing the depth sparsity.

The D415 system evaluates the depth as a proportional inverse of the pixel disparity from the right IR image to the left IR image, where the pixel disparity is evaluated along the rectified epipolar lines [39]. Such a depth, named the disparity shift, can be varied to modify the acquisition field.

For a given disparity shift, the maximum Z value (MaxZ) is given by the following formula:

(1) MaxZ=focal_length (pixels)×baseline (mm)/disparity_shift

where

(2) $$focal\_length\;\left(pixels\right)=\frac{1}{2}\;\frac{Xres\left(pixels\right)}{\tan\left(\frac{HFOV}{2}\right)}$$

The minimum Z value (MinZ) is defined by the following equation, taking into account that the camera searches in a disparity range of 126 bits:

(3) MinZ=focal_length (pixels)×baseline (mm)/(disparity_shift+126)

**Figure 4:**
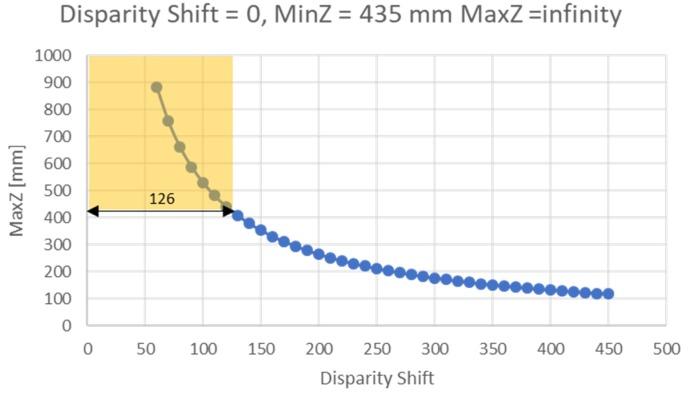
The graph shows the maximum acquirable depth as a function of the disparity shift, calculated considering the HFOV of the camera under investigation (67.24°).

By default, the disparity shift is set to 0 to cover the Z range from 435 mm to infinity. When the disparity shift is increased, the minimum acceptable Z value (i.e., the minimum camera–object distance) decreases, with the consequence of also reducing the maximum Z value. Figure 4 shows MaxZ as a function of the disparity shift, calculated considering the horizontal field of view HFOV of the camera under investigation (67.24°). The yellow rectangle in the figure indicates the range of depth acquired based on the disparity value of the device, which is 126.

As mentioned above, the second parameter used to carry out the sensor characterization consists of the laser power, i.e., the intensity of the infrared pattern projected on the scene to facilitate the search for matches between the left and right images. The depth data is, in fact, generated with stereo vision technology that is optionally assisted by an infrared laser projector. The value of this parameter can vary in the range of 0–360 mW and has a nominal value of 150 mW. If necessary, it can be increased or decreased from the nominal value for better results. For example, if the localized saturation of the laser point is noticed, the power can be reduced; to acquire distant objects, the power of the laser must be increased.

**Figure 5:**
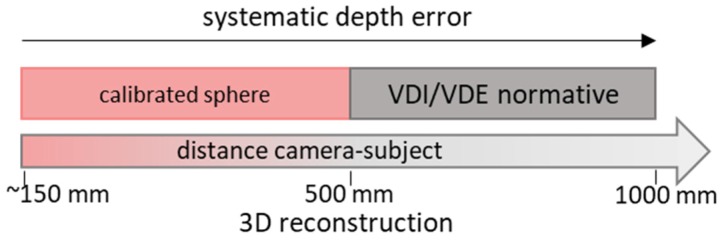
The proposed framework for the characterization of the device: a certified sphere is used in the very-close range (<500 mm); up to 1000 mm, the VDI/VDE 2634 Part 2 standard is applied; the systematic depth errors are evaluated on the entire range; and the 3D reconstruction of an object is tested for a camera–subject distance of 500 mm.

Under the premise of setting the device to its *Default* configuration and of using different values for disparity shift and laser power, the characterization is carried out by following the framework of Figure 5.

In detail, different tests are carried out based on the range under examination:

(a) From very-close object acquisition (150 mm) up to 500 mm, the characterization is assessed using a calibrated sphere positioned at progressive distances from the camera with a fixed pitch of 100 mm. As already mentioned, in this range, the disparity shift is required to be changed.
(b) From 500 mm to 1000 mm, the characterization is performed by following the guidelines of the VDI/VDE 2634 Part 2 standard.
(c) For the entire range, a planarity test is carried out to evaluate systematic depth errors.
(d) With an object–camera distance equal to 500 mm, a test of the camera in allowing the three-dimensional reconstruction of objects with a multi-view acquisition is carried out. Such a reconstruction is made for two different artefacts.

### 3.1. Characterization in the Range 150–500 mm

In the short-range (i.e., from 150 to 500 mm) the VDI/VDE standard cannot be applied due to the fact that the change of the value of disparity shift does not allow the appreciation of the whole working volume required from the standard. In fact, a change in the disparity shift corresponds to a variation of the working volume (which becomes smaller as soon as the target nears the sensor).

Therefore, a different method has been conceived to characterize the device in such a range, inspired by Reference [33]. In particular, a calibrated sphere with a certified diameter of 25.4 mm was used to assess the camera’s performance. The short-range was ideally divided into sub-regions with a 100 mm pitch (with the exception of the range 150–200 mm), thus defining 4 operative ranges where the acquisition is performed (see Table 5). Starting from a distance between the calibrated sphere and the sensor equal to 150 mm, the sphere is acquired at increasing distances. For each operative range, the disparity shift can be varied spanning from a given minimum and maximum value to obtain the correct acquisition of the sphere, as stated in Equations (1) and (3). This allows the definition of a disparity shift range for each operative range (see second column of Table 5).

**Figure 6:**
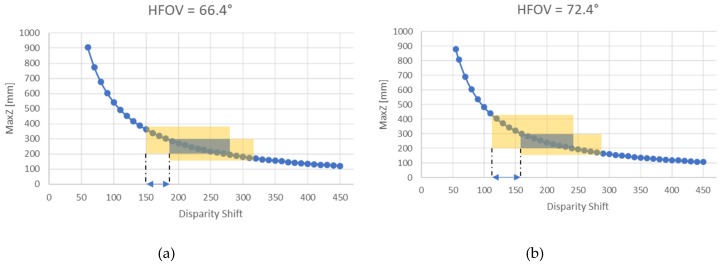
The graph shows the maximum acquirable depth as a function of the disparity shift, calculated considering HFOV = 69.4° ± 3°. The blue rectangle indicates the depth range 200–300 mm. The yellow rectangles are obtained computing the first and the last values of disparity shift that allow the acquisition of the whole sub-range, which are 150–180 when HFOV = 66.4°, and 114–160 when HFOV = 72.4°.

The values shown in Table 5 specify the range of disparity shift that allows the acquisition in a selected depth window. The values have been calculated considering the two extremes of the HFOV as indicated in the specifics (i.e., 69.4° ± 3°). Figure 6 shows an example of the calculation of the disparity shift range when considering the sub-range of depth 200–300 mm.

Figure 6a shows the case in which the HFOV is equal to 66.4°, in which the resulting range of the disparity shift is 150–180; if the HFOV is instead equal to 72.4° (Figure 6b), the resulting range of the disparity shift is 114–160. Consequently, if a disparity shift value between 150 and 160 is used, in both configurations, the entire sub-range can be acquired.

Any value for the disparity shift falling within the disparity shift range can be selected to perform the acquisition in a given sub-region. Therefore, the preferred values are the ones listed in third column of Table 5. Using this configuration, only the laser power remains as a changing parameter to test the device performance. In addition to the nominal value, the values 0, 250 and 360 mW were tested.

**Figure 7:**
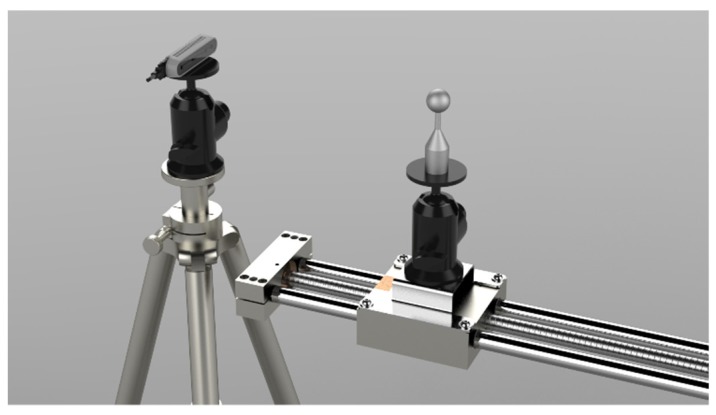
The acquisition setup for the very-close range evaluation test: the camera is mounted on a tripod facing the linear guide on which the sphere moves.

To carry out the test, the camera was mounted on a stable tripod and the sphere was fixed on a sliding linear guide having a 4 mm pitch (see Figure 7).

**Figure 8:**
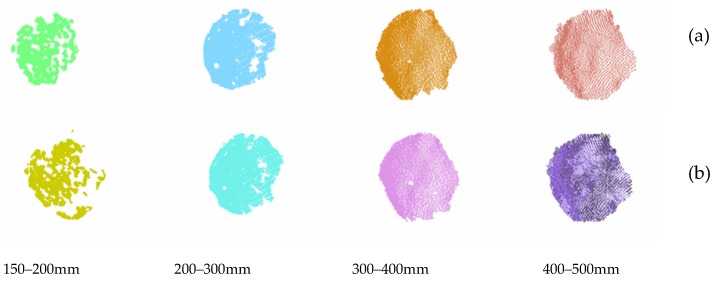
(**a**) The point clouds of the sphere acquired at increasing distance with maximum laser power (360 mW); (**b**) the point clouds of the sphere acquired at increasing distance with a laser power of 250 mW.

As noted, the calibrated sphere was scanned every 10 cm and, in each sub-region, 4 different laser power levels were tested (i.e., 0, 150, 250, and 360 mW). In Figure 8, 2 examples are reported out of the entire test set; respectively, Figure 8a shows the sphere acquired at increasing distance with the maximum laser power (360 mW), and Figure 8b shows the sphere acquired at increasing distance with a laser power equal to 250mW.

Each acquisition was processed by extracting the best-fit sphere and comparing the obtained diameter (D) with the ground truth diameter (D_gt_ = 25.4 mm), thus defining the error E as follows:

(4) $$E=\left|D_{\mathrm{gt}}-D\right|$$

**Figure 9:**
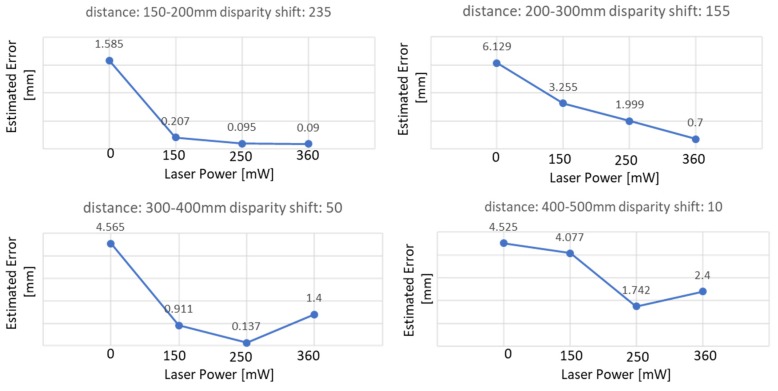
The errors in the estimation of the calibrated sphere for each sub-range from 150 to 500 mm, evaluated for increasing the value of the laser power (0 to 360 mW)

Figure 9 shows the error value obtained for each sub-region with different values of the laser power parameter; such an error decreases as the laser power increases.

According to experimental results, in the very-close range of 150–500 mm, the biggest error corresponded to a laser power of 0. When the laser power used was the default (i.e., 150 mW), the errors obtained in the reproduction of the geometry of the sphere, intended as the difference between the actual diameter and the estimated diameter, ranged from approximately 0.2 mm to 4 mm, with an average error of 2.11 mm for the considered value of the disparity shift. As can be seen from the graphs shown in the figure, the error decreases if the laser power value does not exceed 250 mW and can be reduced by up to 20%. For higher values, the error behavior may vary, probably due to the laser-speckle effect, i.e., the coherent light interaction with the reference plane. In fact, scattering can create photometric inconsistencies producing matches where there are none [18]. From the results of the performed test, the scattering effect can be observed in the acquisition range of 300–500 mm when the laser power is equal to 360 mW. Globally, the average error obtained with a laser power equal to 250 mW was 0.99 mm, compared to an average error of 1.15 mm obtained with laser power equal to 360 mW.

### 3.2. Characterization in the Range 500–1000 mm; VDI/VDE Standard

The range of 500–1000 mm was characterized following the VDI/VDE 2634 Part 2 recommendations in order to provide a comparison with the previous Intel camera model (the Intel RealSense SR300) and other RGB-D devices, as mentioned in Section 1. In the first instance, the diagonal of the working volume L_0_ was determined; as mentioned above, this parameter is needed for sizing the spheres, the bar and the plane for the performance evaluation test. In this regard, the truncated pyramid representing the working volume in the range of 500–1000 mm has a diagonal L_0_ equal to 1291.9 mm. According to the standard, the sphere used to characterize the probing error must have a diameter between 0.1 and 0.2 times L_0_. This translates into a diameter between 129.19 mm and 258.38 mm.

The distance between the centers of the two spheres of the ball-bar (to characterize the sphere-spacing error) is suggested to be greater than 0.3 times L_0_; accordingly, in the present test, such a distance is equal to 387.57 mm. Finally, the plane for the flatness error should be longer than 0.5 times L_0_, i.e., greater than 645.95 mm.

**Figure 10:**
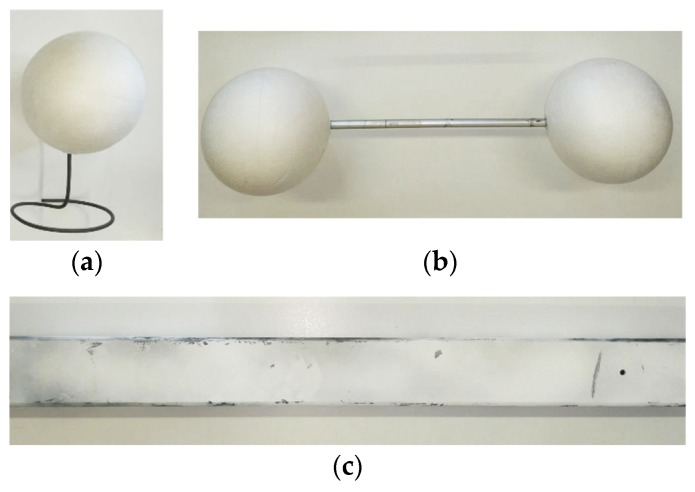
The artefacts proposed to measure the quality parameters inspired by the VDI/VDE standard: (**a**) single sphere having a diameter of 143.15 mm; (**b**) ball-bar composed of two spheres fixed at 391.58 mm from one another; and (**c**) iron bar of dimensions 50 × 646 mm^2^.

Starting from the values suggested by the standard, the artifacts chosen for carrying out the test had the following dimensions: the single sphere had a diameter of 143.15 mm, the distance between the centers of the two spheres of the ball-bar was 391.58 mm, and the plane for the flatness error was 646 mm long (Figure 10). These values were measured using a high precision scanner, the Romer Absolute Arm 7520 SI/SE (Hexagon Metrology S.p.A., Turin, Italy), which has an accuracy of ±0.063 mm, and therefore allowed for sufficiently reliable measurements for the purpose of this work.

As mentioned in Section 1, the following errors have to be measured to assess the accuracy of the imaging device.

**Figure 11:**
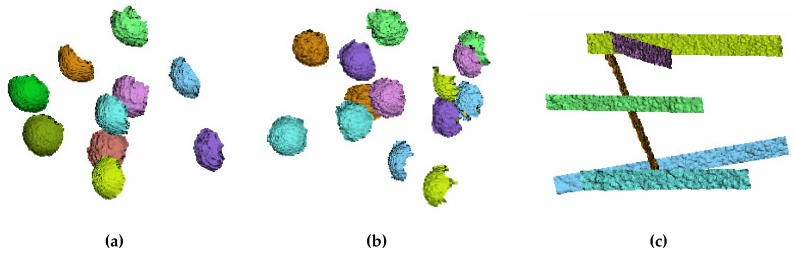
(**a**) The 3D recording of the single sphere captured in 10 arbitrary positions homogeneously distributed within the working volume. (**b**) 3D points of the ball-bar captured in 7 positions within the working volume (the same sphere color corresponds to the same bar). (**c**) 3D points of the plane captured in 6 positions within the working volume.

The form probing error (PF) is defined as the absolute average value of the radial distances between the real measured points and a best-fit sphere evaluated according to the least-squares method, obtained for a number of acquisitions. The size probing error (PS) is the absolute average value of the differences between the estimated and the “true” diameter of the sphere, again obtained using several acquisitions. To perform the evaluation of both probing errors, the target sphere has been positioned in 10 sequential arbitrary locations (as indicated by the standard) within the working volume (Figure 11a), thus defining 10 different values for each error. Accordingly:

(5) $$PF_{i}=\left|R_{imax}\;–\;R_{imin}\right|$$

where $PF_{i}$ is the error measurement for the *i*th acquisition, with i=1:10 and $R_{imax}$ and $R_{imin}$ are, respectively, the maximal and minimal distances of the measured surface points of the *i*th sphere from the center of the compensating element. Therefore, the form probing error is given by

(6) $$PF=\frac{\sum_{i=1}^{10}PF_{i}}{10}$$

Additionally, for the size probing error, it is possible to define the *i*th measurement:

(7) $$PS_{i}=\;\left|D_{mi}-Dc\right|$$

where $D_{mi}$ is the measured diameter of the sphere acquired at the *i*th position and Dc is the actual diameter of the calibrated sphere.

Therefore, it is possible to evaluate the size probing error according to the following equation:

(8) $$PS=\frac{\sum_{i=1}^{10}PS_{i}}{10}$$

The sphere spacing error (SS) is measured by using the ball-bar target. SS is the absolute average value of the differences between the acquired distance and the “true” distance (lk) between the centers of the two spheres (estimated from the point cloud data using a best-fit sphere fitting) for a number of different acquisitions. The target ball-bar was therefore positioned in 7 sequential arbitrary locations (as indicated by the standard) within the working volume (Figure 11b). For each acquisition it is possible to evaluate the *j*th error $SS_{j}$:

(9) $$SS_{j}=\left|l_{mj}-lk\right|$$

As a consequence:

(10) $$SS=\frac{\sum_{j=1}^{7}SS_{j}}{7}.$$

According to the VDI/VDE standard, the flatness (or planarity) error (F), is computed as the average of the flatness $F_{k}$ obtained for the acquisition of the iron bar positioned in 6 arbitrary positions within the working volume. In detail, $F_{k}$ is defined as the distance between the two parallel planes enclosing the *k*th point cloud. Consequently:

(11) $$F=\frac{\sum_{k=1}^{6}F_{k}}{6}$$

The values obtained by calculating the errors proposed by the standard are shown in Table 6, along with the results obtained with the Intel RealSense SR300, the Kinect v2 and the PrimeSense Carmine 1.09. Such results were obtained in Reference [35] by repeating the test setup provided by the standard for competitor cameras. As with the previous model, this experiment has been carried out on both raw and filtered data. To obtain the latter, the post-processing function of the SDK was activated, which by default applies decimation, spatial, time and edge-preserving filtering.

Referring to the probing form error (PF), the new Intel device performances are comparable to the PrimeSense Carmine 1.09 and SR300, while Kinect v2 is characterized by a higher error. If the probing size error PS is considered, the Intel devices with filtered data prove to be the most effective, and the Kinect v2 performs analogously to the SR300 and D415 with raw data. The higher performance of both the D415 and SR300 Intel devices is even more evident when dealing with the sphere spacing error SS; in fact, the $\Delta_{SS}$ is considerably higher for tested competitor cameras. The performance in terms of flatness error F is almost the same for the SR300 and the D415 with filtered data and the PrimeSense Carmine 1.09. Interestingly, the behavior of the Kinect v2 in all tests is quite satisfying considering that this camera system is specifically designed to work in the medium to long range. It is interesting to note that the sphere spacing error measured for the SR300 and D415 using row data is almost the same assessed using optimized settings. This may be because the moderate smoothing effect obtained using the optimized setting does not have a particular effect on the position of the sphere’s center when compared with the same position evaluated using raw data. When comparing the two RealSense models, it is noted that the results obtained are comparable: if the raw data are considered, the new camera shows a slightly better performance for the whole test-set; on the other hand, the results obtained with filtered data are similar.

As reported in Reference [35], the discussion can be extended to other results from scientific literature. Reference [40] reports the results computed for the probing errors PF and PS) with several cameras, among them the Occipital Structure Sensor device, which is one of the most relevant competitors of Intel RealSense devices. This sensor scored an average value of ∼10 mm for PF and ∼2.2 mm for PS, while the values obtained with the latest Intel model for PF are 13.83 mm for raw data and 8.42 mm for filtered data, and for PS, 3.5 mm and 1.91 mm, respectively.

Regarding the flatness error, F, the performance of the D415 can be compared to the Occipital Structure Sensor device [40] with the result of reducing the error up to 50% when using D415 with filtered data.

The sphere spacing error, SS, can instead be compared to the results in Reference [41] obtained with the Asus Xtion Pro camera, whose results spanned from −8 to 2 mm compared to ∼5 mm obtained with the D415 model.

### 3.3. Systematic Depth Errors in the Entire Range 150–1000 mm

**Figure 12:**
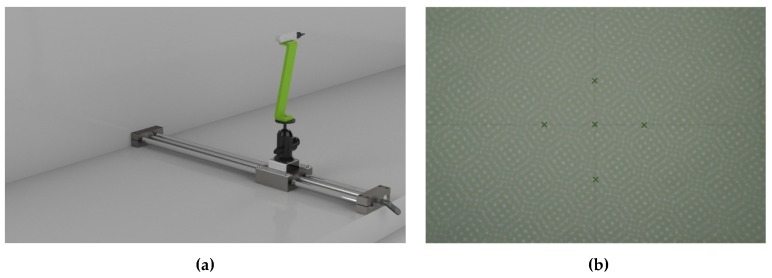
(**a**) The acquisition setup for systematic depth error evaluation test: the camera is mounted on a linear guide perpendicular to a planar surface. (**b**) Example of left camera frame acquisition of the reference plane with the physical marker.

As also reported in Reference [35], one of the most common systematic errors for RGB-D devices is the so-called “inhomogeneous distance”, i.e., the set of possible errors that may result from acquisitions at different distances from the sensor. To study these errors, the camera is required to be positioned perpendicularly to a surface plate and to acquisition at incremental distances (see Figure 12a) in the range 200–1000 mm with a pitch of 100 mm.

To this aim, the first step consists of positioning a linear guide perpendicular to the reference plane (see Figure 12a). In particular, the guide is iteratively positioned to (as closely as possible) reach the perpendicularity condition between the axis of one of the cylindrical rails and the reference plane. In the final orientation, the angle between the cylindrical rail and the reference plane, measured by using the Romer Absolute Arm 7520 SI/SE equipped with its contact probe, was 90.08°.

Once the correct positioning of the guide was assured, a dot IR reflective marker was physically placed on the reference plane. Subsequently, the camera was positioned so that the principal point of the left IR camera (which coincides with one on the depth map, plotted on the depth map image with a green cross) superimposed onto such a marker when the camera, mounted on the linear guide, moved away from the plane. In detail, the camera orientation was refined by assuring that for the two frames, acquired respectively at 200 mm and 1000 mm, the distance between the principal point and the acquired marker was lower than two pixels. The principal point was evaluated by performing an intrinsic calibration [42]. Although the depth map rotation around the optical axis of the left IR camera did not affect the metrological assessment of the systematic depth error, a rough alignment of the X and Y image axes with the horizontal and vertical directions was sought. Accordingly, two additional orthogonal lines were physically drawn on the reference plane (see Figure 12b) in the horizontal and vertical directions. Line orientation was checked by means of a laser level. The rough orientation of the camera was obtained by visually superimposing four additional crosses plotted on the left IR camera image on the drawn lines. Finally, the pitch of the linear guide, equal to 4 mm (therefore known a priori), assured the plane–camera distance.

The aim was to assess two types of errors: systematic non-planarity errors and offset errors on the depth. The first one was evaluated by referring to a ground truth plane (with a certified flatness of 50 μm), as in Reference [35], to verify the flatness of the obtained scan. The latter was analyzed by studying the distance in Z between the obtained scan and the ground truth plane.

**Figure 13:**
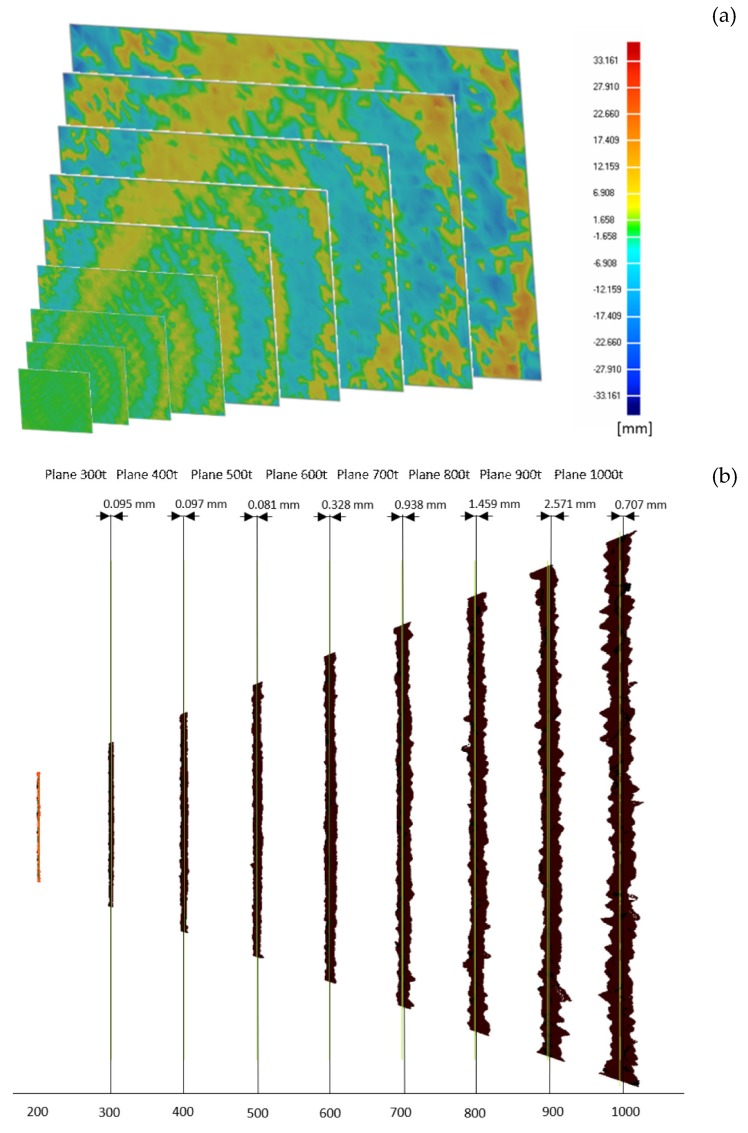
(**a**) The rrror map between the target plane and the 9 point clouds acquired using the D415 device with increasing distance from the plane itself. (**b**) Evaluation of the depth-offset error for the 9 acquired planes.

Figure 13 shows the results obtained for the two tests. In detail, Figure 13a shows the error map between the scanned data and the best fitting planes built on such data, to highlight the non-planarity error that is introduced by the camera. The average deviation of the point clouds with respect to the ground truth planes ranges from 0.004 mm with a standard deviation of 0.345 mm at a distance of 200 mm from the plane, up to 0.237 mm with a standard deviation of 4.74 mm at a distance of 1 m. The maximum recorded error value spans from 1.6 mm to 26.5 mm, at 200 mm and 1000 mm distance, respectively. Figure 13b shows, instead, that nearly no error is present with regard to the depth offset, i.e., the point clouds are almost correctly positioned at a distance of 100 mm from each other. In fact, the best fitting planes evaluated for each point cloud using the Geomagic Design X^®^ software package (3D Systems, Rock Hill, SC, USA) show a distance from the ground truth plane with a minimum value of 0.06 mm and a maximum value of 2.54 mm for the planes acquired at distances of 1000 mm and 900 mm, respectively.

The analysis of systematic depth errors led to interesting results, especially when compared to those obtained for the SR300. In the first instance, compared to the previous model, both the non-planarity of the single acquisitions and the depth offset have improved considerably. Specifically, as shown in Figure 13b, there is nearly no offset between the acquired data and the best fitting planes; moreover, the clouds do not have the “twist effect” that was noticed of the previous model. Noise tends to be distributed locally, leading to higher peaks than those reported by the previous sensor; a non-linearity, probably introduced by the lenses [33], can also be noted.

### 3.4. 3D Object Reconstruction 

The last test carried out concerns the three-dimensional reconstruction of objects. Inspired by Part 3 of the VDI/VDE 2634 standard [43], which provides guidelines for the positioning of the camera around the artifact, the object under examination was rotated with respect to a stable camera.

**Figure 14:**
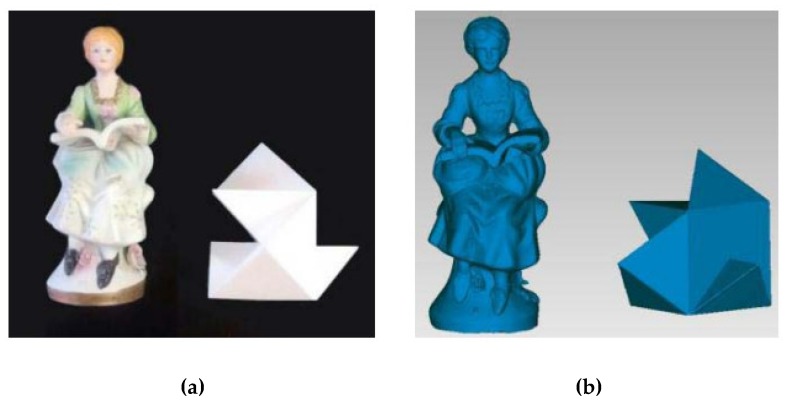
(**a**) The test objects for multiple-view reconstruction: a freeform statue and a 3D tangram; (**b**) reference 3D models for multiple-view reconstruction, obtained starting from the Romer Absolute Arm acquisition (an accuracy of ±0.063 mm).

It was decided to evaluate the accuracy of the reconstruction with the same two objects used to characterize the SR300 in [35]: a freeform smooth object (∼200 mm high and ∼70 mm wide) and a 3D tangram object (bounding box with dimensions of 150 mm high and 150 mm wide). Again, to provide an effective and easily replicable evaluation, the camera was used in the default configuration, with the only modified parameter being the depth unit, set to the best achievable resolution. To ensure the best performance, the camera–object distance was set to the smaller achievable value of approximately 500 mm. To obtain two reference models for assessing the device performances, the Romer Absolute Arm 7520 SI/SE was used (see Figure 14). The meshes, obtained using the Geomagic^®^ Studio software package starting from the point cloud acquired with the D415 camera, and the reference 3D model were globally registered using the iterative closest point ICP algorithm [44]. To increase the quality of the mesh reconstruction, 5% of the borders were removed prior to performing the alignment.

**Figure 15:**
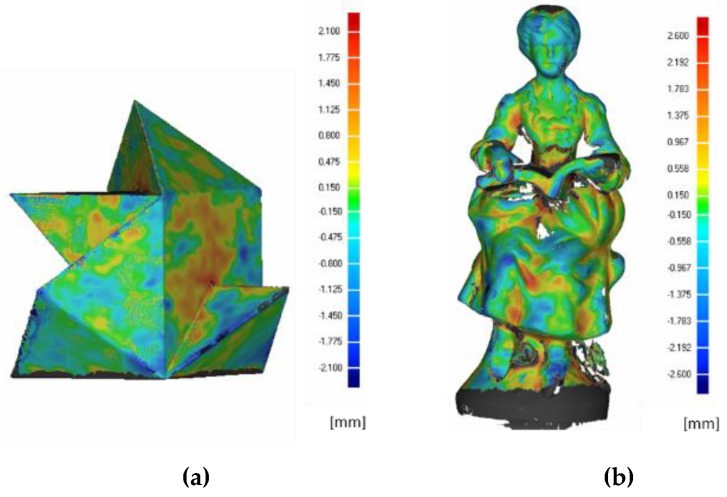
(**a**) The 3D comparison between the tangram ground truth and the D415 acquired data. Errors are in mm. (**b**) The 3D comparison between the statue ground truth and the D415 acquired data. Errors are in mm.

Figure 15 shows the Euclidean distances between the acquired artifacts and the ground truth. The comparison between the ground truth and the acquired data is limited to the target portion obtained with the multiple-view acquisition; grey areas in Figure 15 are not, therefore, considered. As far as the tangram (Figure 15a) is concerned, an average deviation of 0.110 mm is obtained with a standard deviation of 0.902 mm; for the statue (Figure 15b), the average deviation obtained is −0.029 mm with a standard deviation of 1.856 mm.

The multi-view acquisition test for the full 3D reconstruction proves (see Figure 15) that both geometries are fairly measured, particularly if it is considered that for other RGB-D devices (e.g., the Microsoft Kinect) it has been evaluated that 3D reconstruction error can span an interval of 27 mm [36]. It can also be noted that there is no substantial difference with regard to the error assessed in the two reconstructions of the prismatic shape (tangram 3D) and the free form shape (statue), when the latest Intel model is used. Furthermore, the results shown here can be compared with the results obtained by scanning the same objects with the SR300 (Table 7). It can be noted that the results are fully comparable, with the main difference found in the reconstruction of the statue which has, as noted, a free form shape: the new model performs with an average error of −0.029 mm against −0.2 mm obtained with the previous model.

## 4. Discussion and Conclusions

Given the growing popularity of RGB-D devices mainly due to their versatility and the interest that Intel RealSense devices have obtained in recent years for their accuracy, compactness and ease of use, in this work, a metrological evaluation of the latest model presented by Intel, the RealSense D415, was carried out. After investigating the operating principles of this device, a panel of tests was defined based on the distance between the camera and the examined scene. The main objective was, other than characterizing the new model, to compare this device with the previous model presented by Intel, the RealSense SR300 and, where possible, with other competitor RGB-D cameras.

In this work, the devices were compared through four types of tests: the error measured using a calibrated sphere at a very close range, the errors measured with the VDI/VDE 2634 Part 2 standard, the systematic depth errors extracted with the acquisition of a planar surface at increasing distances, and the 3D reconstruction of an object.

Tests have shown that the new D415 model is fully comparable to its predecessor model in terms of errors assessed through the VDI/VDE standard. The device is also in line with the results obtained with other devices in the scientific literature and exceeds their performance when considering the filtered data.

As for the 3D reconstruction, a comparison can be made with the previous Intel model, since both of them work in the close range with the result of a comparable deviation error.

The most interesting result is obtained from the estimation of the systematic depth errors: the D415, in fact, reports better results, both in terms of average flatness error and of displacement in Z from the real plane, with a maximum displacement from the ground truth plane of 2.52 mm.

In addition, the device is sufficiently accurate when acquiring very close distances (e.g., from 150 mm to 500 mm) as the reconstruction error in this range can be 0.99 mm on average when using a laser power equal to 250 mW.

As demonstrated by the experimental results of the characterization, it is possible to state that although the device is designed for addressing applications such as tracking, gaming or gesture recognition, it could also be satisfactorily employed as a 3D scanner, i.e., it could be used as a low-cost device for a number of 3D-scanning applications in a number of fields, including, among others, health, fashion, fitness and cultural heritage.

## Figures and Tables

**Table 1:** The technical specifications of Intel RealSense D415.

Environment	Indoor and outdoor
Depth Technology	Active infrared (IR) stereo
Image Sensor Technology	Rolling shutter: 1.4 μm × 1.4 μm pixel size
Depth Field of View (FOV) (Horizontal × Vertical) for HD 16:9	69.4 × 42.5° (±3°)
Depth Stream Output Resolution	Up to 1280 × 720 pixels
Depth Stream Output Frame Rate	Up to 90 fps
Minimum Depth Distance (Min-Z)	0.16 m
Maximum Range	~10 m
RGB Sensor Resolution and Frame Rate	Up to 1920 × 1080 pixels at 30 fps
RGB Sensor FOV (Horizontal × Vertical)	69.4 × 42.5° (±3°)
Camera Dimension (Length × Depth × Height)	99 mm × 20 mm × 23 mm
Connector	USB Type-C

**Table 2:** The point density and framed area variation at increasing camera–scene distance, computed using the FOV indicated in the Intel specifications (69.4 × 42.5°).

Distance (mm)	Scale Factor	X (mm)	Y (mm)	Framed Area (mm^2^)	Point Density (point/mm^2^)
500	0.541	692.4	388.9	269,272.400	3.423
600	0.649	830.9	466.7	387,752.256	2.377
700	0.757	969.4	544.4	527,773.904	1.746
800	0.866	1107.9	622.2	689,337.344	1.337
900	0.974	1246.4	700.0	872,442.576	1.056
1000	1.082	1384.9	777.8	1,077,089.600	0.856
1250	1.352	1731.1	972.2	1,682,952.501	0.548
1500	1.623	2077.3	1166.6	2,423,451.601	0.380

**Table 3:** The point density and framed area variation at increasing camera–scene distance, computed using maximum and minimum FOV variations (69.4 × 42.5° +/−3°).

	FOV – 3°					FOV + 3°				
Distance (mm)	Scale Factor	X (mm)	Y (mm)	Framed Area (mm^2^)	Point Density (point/mm^2^)	Scale Factor	X (mm)	Y (mm)	Framed Area (mm^2^)	Point Density (point/mm^2^)
**500**	0.874	745	419	312,607.4	0.98	0.748	638	359	229,168.6	1.34
**600**	1.048	895	503	450,154.7	0.68	0.898	766	431	330,002.8	0.93
**700**	1.223	1044	587	612,710.6	0.50	1.047	894	503	449,170.5	0.68
**800**	1.398	1193	671	800,275.0	0.38	1.197	1021	574	586,671.6	0.52
**900**	1.573	1342	755	1,012,848.1	0.30	1.346	1149	646	742,506.3	0.41
**1000**	1.747	1491	839	1,250,429.7	0.25	1.496	1277	718	916,674.4	0.34
**1250**	2.184	1864	1048	1,953,796.4	0.16	1.870	1596	898	1,432,303.8	0.21
**1500**	2.621	2236	1258	2,813,466.8	0.11	2.244	1915	1077	2,062,517.5	0.15

**Table 4:** The point density and framed area variation at increasing camera–scene distance, computed using the FOV of camera under investigation (67.24 × 41.01°).

Distance (mm)	Scale Factor	X (mm)	Y (mm)	Framed Area (mm^2^)	Point Density (point/mm^2^)
500	0.779	665	374	248,745.7	3.70
600	0.935	798	449	358,193.9	2.57
700	1.091	931	524	487,541.6	1.89
800	1.247	1064	599	636,789.1	1.45
900	1.403	1197	673	805,936.2	1.14
1000	1.559	1330	748	994,982.9	0.93
1250	1.714	1463	823	1,203,929.3	0.77
1500	1.870	1596	898	1,432,775.4	0.64

**Table 5:** The tested value of disparity shift and laser power for each sub-range.

Operative Range (mm)	Disparity Shift Range	Selected Disparity Shift Value	Laser Power (mW)
150–200	234–240	235	0, 150, 250, 360
200–300	150–160	155	0, 150, 250, 360
300–400	49–125	50	0, 150, 250, 360
400–500	9–95	10	0, 150, 250, 360

**Table 6:** The comparison between D415, SR300, Kinect v2 and PrimeSense Carmine 1.09 in terms of VDI/VDE error assessment.

Device	D415 (Raw Data)	D415 (Filtered Data)	SR 300 (Filtered Data)	SR300 (Raw Data)	Kinect v2	Carmine 1.09
PF (mm)	13.83	8.42	8.30	15.43	20.13	9.32
PS (mm)	3.50	1.34	1.91	4.57	3.87	9.41
SS (mm)	4.99	5.03	6.05	5.11	19.7	26.08
F (mm)	15.36	9.45	6.88	19.33	12.58	6.71

**Table 7:** The average deviation (avg) and standard deviation (std) from the ground truth data obtained for the 3D reconstruction of the two artifacts for D415 and SR300 cameras.

	Reconstruction Deviation from Ground Truth	
	Tangram	Statue
**D415**	avg: 0.11 mm, std:0.90 mm	avg: −0.029 mm, std:1.85 mm
**SR300**	avg: 0.10 mm, std: 0.4 mm	avg: −0.2 mm, std: 1.01 mm
